# Enterocin: Promising Biopreservative Produced by *Enterococcus* sp.

**DOI:** 10.3390/microorganisms10040684

**Published:** 2022-03-23

**Authors:** Melisa Elsie Kasimin, Suriyani Shamsuddin, Arnold Marshall Molujin, Mohd Khalizan Sabullah, Jualang Azlan Gansau, Roslina Jawan

**Affiliations:** Biotechnology Programme, Faculty of Science and Natural Resources, Universiti Malaysia Sabah, Jalan UMS, Kota Kinabalu 88400, Sabah, Malaysia; leyssa145@gmail.com (M.E.K.); suriyanishamsuddin8@gmail.com (S.S.); arnold.molujin@gmail.com (A.M.M.); khalizan@ums.edu.my (M.K.S.); azlanajg@ums.edu.my (J.A.G.)

**Keywords:** food preservation, natural preservatives, lactic acid bacteria, antimicrobial substances, enterocin, commercial techniques

## Abstract

Food preservation is a method used to handle and treat food products to slow down food spoilage and subsequently reduce the risk of foodborne illness. Nowadays, the demand for natural preservatives over chemical preservatives in food is increasing due to the awareness of consuming healthy food products without the risk of harmful side effects. Thus, the research and development of preservation techniques, referred to as biopreservation, is growing rapidly. In biopreservation methods, microorganisms that are known as lactic acid bacteria (LAB) and their antimicrobial substances are used to extend shelf life and maintain the nutritional value of foods. Among the most studied LAB are from the genus *Enterococcus*, which produces a bacteriocin called enterocin. Bacteriocins are ribosomal-synthesized antimicrobial peptides that are capable of inhibiting the growth of pathogenic bacteria that cause spoilage in food. LAB is generally regarded as safe (GRAS) for human consumption. The current application of LAB, notably *Enterococcus* sp. in the biopreservation of meat and meat-based products was highlighted in this review. This report also includes information on the effects of enzymes, temperature, and pH on the stability of bacteriocin produced by *Enterococcus* sp. An extensive compilation of numerous industry procedures for preserving meat has also been emphasized, highlighting the benefits and drawbacks of each method.

## 1. Introduction

Food preservation is synonymous with its use in the food industry. Food preservatives are applied in food to prolong the shelf life and improve the quality of food products. Failure in applying this technique will cause a huge impact on human health. Commonly, food made from animal sources is highly perishable as they have high nutrients, moisture, and a neutral pH [1]. Various food preservation techniques have been used, such as traditional and modern preservation techniques. In this modern era, traditional preservation techniques have been less applied by the food industry as they often alter and eliminate the nutrients of the product. Consequently, modern techniques for food preservation have started to gain attention, such as the use of beneficial bacteria to maintain and improve the quality of the product. Such techniques are also capable to extend shelf life through the application of protective microbes known as lactic acid bacteria (LAB) [2]. LAB is a group of heterogeneous bacteria that play an important role in the food industry as a preservative agent in various food products, such as kimchi, bread, cheese, sausages, and fermented meat [3]. Biopreservation is a technique for extending the shelf life of food by using microbes or safe antimicrobials produced by LAB. These bacteria exhibit their antimicrobial properties, which retain the unique taste and texture of the food products. The main compounds produced by these bacteria are bacteriocin, organic acids, and hydrogen peroxide [1].

The Food and Agriculture Organization of the United Nations has expressed concern over the production of agricultural food to accommodate the growing world population, which will have a detrimental impact on humans in terms of health, safety, and quality. If this problem is mismanaged, 30 to 40% of food manufacturing will be wiped out due to pests and several other factors that may lead to a paralyzed economy [4]. The control of pests and pathogens in food products nowadays has become more challenging due to the widespread use of artificial ingredients. The public is aware of the danger of using artificial ingredients in food, thus, demand for food products that are high in quality, safe, and use natural ingredients is increasing.

Therefore, to curb the problem of foodborne illnesses from continuing to pose threat to human health, an initiative to study the ability of LAB against spoilage bacteria has started to gain more attention. LAB plays a crucial role in the agricultural and clinical sectors and has been widely used in the production of food products. Yusra and Effendi [5] found that the LAB called fermenting bacteria have often been used as a preservative agent in commercial meat, vegetable and fruit, beverage, and dairy products. These food preservatives are intended to control the growth of pathogens, spoilage bacteria, yeast, and spoilage fungi. These bacteria are naturally found in the respiratory organs, intestines, and reproductive organs of humans and animals. The common genus of LAB includes *Lactobacillus*, *Leuconostoc*, *Pediococcus*, and *Streptococcus*, while the genus of LAB that has been often found in food includes *Aerococcus*, *Carnobacterium*, *Lactococcus*, *Onecoccus*, *Tetragenococcus*, *Veganococcus*, and *Weisella* [6].

**Figure 1:**
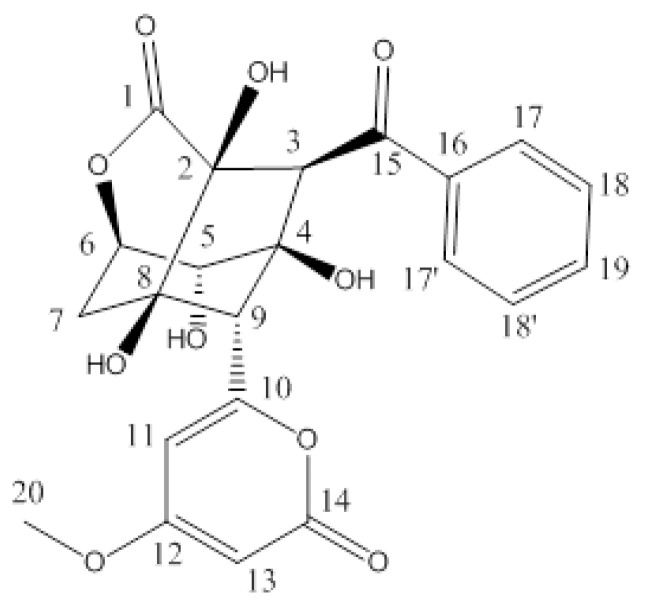
Chemical structure of enterocin.

A study on the potential use of *Enterococcus* sp. as a food preservative has been actively pursued due to its ability to produce a bacteriocin known as enterocin [7]. Enterocin is a type of bacteriocin that consists of peptides (Figure 1) with a small molecular weight and has specific antimicrobial activity that can inhibit the growth of Gram-positive and spoilage bacteria [8]. According to Hanchi et al. [7], enterocin has a specific antimicrobial activity to inhibit the growth of species that are closely related to the producing bacteria and capable to inhibit the growth of Gram-positive pathogens, such as *Listeria monocytogenes*. Such ability has prompted its use as a starter and protective culture in the food preservation process [9].

## 2. Classification of Enterocin

Enterocin is a bacteriocin produced by *Enterococcus* sp., such as *E. faecalis*, *E. faecium*, *E. durans*, *E. mundtii*, *E. lactis*, and several others. The majority of these species are isolated from food, including dairy products, sausages, vegetables, and raw materials [10]. The first successfully purified enterocin was enterocin As-48 produced by *E. faecium*, which has been classified as a cyclic peptide antibiotic. The finding has subsequently led to the discovery of a new enterocin-producing *Enterococcus* [8].

Enterocin is a cationic peptide synthesized by ribosomes and has the characteristics of a compound with polar and hydrophobic regions due to the presence of excess lysyl and arginyl residues [11]. It also has a heat-stable peptide with a molecular weight of about 20–60 amino acids, insensitive to rennet and stable at varying pH values [9]. Enterocin can be classified into four classes namely the lantibiotic (Class I), non-lantibiotic (Class II), cyclic enterocin (Class III), and enterocin with high molecular weight (Class IV). Most enterocin produced by *Enterococcus* sp. are categorized as class II as shown in (Table 1).

Enterocin in class I is a lantibiotic with peptides that undergo enzymatic modification during the process of biosynthesis, forming unusual amino acid molecular structures. The modification affects the properties of the enterocin, such as lantibiotics, Beta-methyl lanthionine, and dehydrated residues. The class I enterocin consists of peptide leaders that are important for enzyme recognition, transport, and ensuring that peptides remain in an inactive state which coalesces into core peptides [10].

The structure of the peptide of class II enterocin is typically unmodified, conferring the ability to develop into a mature form without requiring enzyme, peptide leaders, or carriers. Class II enterocin consists of hydrophilic and cationic regions with common sequences of YGNGV (tyrosine, asparagine, glycine, and valine) at the N-terminal end and disulfide bridges formed by two cysteines at the N-terminal end [12], which participate in the interaction with targeted bacteria. For instance, pediocin PA-1 is known as a bacteriocin of class II, whose properties are similar to that of enterocin class II. According to Ennahar et al. [13], the class II peptide has a strong inhibitory effect against *L. monocytogenes* in food. The presence of hydrophobic and cationic regions with an orderly sequence of YGNGV attach to the cytoplasmic membrane of the targeted bacteria, allowing pediocin molecules to penetrate the membrane and form pores that result in cell death [12]. Enterocin is produced by the majority of *Enterococcus* sp. in class II. According to Ben Braïek and Smaoui [9], enterocin A is the most potent antimicrobial bacteriocin in the class, whereby it is also produced together with other bacteriocins, such as enterocin B, P, L50, or Q. This is proven with the ability of *E. lactis* to produce three types of enterocin namely enterocin A, B, and P. Class III enterocin is heat-labile and cyclic and includes enterocin As-48. Enterocin As-48 produced by *E. faecalis* contains a large number of basic amino acids as compared to acidic residues, with most of the amino acids being hydrophobic (Ala, Pro, Val, Met, Ile, Leu, and Phe) and hydrophilic (Ser, Gly, Thr, and Tyr). Enterocin As-48 has 70 amino acid residues in total and contains non-modified amino acid residues or disulfide bridges [14]. Class IV enterocin includes enterolysin A, which has a molecular weight between 34 and 501 amino acids, and is heat-labile, has a broad inhibitory spectrum and differs from other enterocins of other classes, which disrupt the cell wall of sensitive bacteria causing the cell to lyse. Not only that, the catalytic domains of various cell wall-degrading proteins with modular structures are homologous to the N terminus of enterolysin A [15]. Generally, the majority of the enterocin produced by *Enterococcus* sp. are categorized in class II because they have an N-terminal structure that influences the level of antimicrobial and capability of producing anti-listerial activity.

**Table 1:** Enterocin classification.

Class	Sub-Class	Sub-Class/Characteristics	Example	References
I	Lantibotic enterocin	-	Cytolysine	[10]
II	Class II-1: Consists of hydrophilic and cationic regions with a consensus sequence of YGNGV located at the end of N terminus and sulfide bridge formed by two cysteine residues at the extremity of N terminus	Sub-group 1: Possess the ABC transport system for the secretion of enterocin	Enterocin L50A, mundticin	[7,9,14,16]
		Sub-group 2: Bacteriocin production through pre-mature proteins	Enterocin P, Enterocin M, Enterocin B	
	Class II-2: The synthesis process occurs without leader peptides, devoid of consensus sequence as well as the secretion of the ABC transport system.	Sub-group 1: Composed of monomeric protein	Enterocin RJ-11, Enterocin Q, Enterocin EJ97	
		Sub-group 2: Required in the formation of the heterodimeric complex	Enterocin L50, Enterocin MR10	
	Class II-3	Enterocin-like linear non-pediocin	Enterocin B, Durancin GL	
IIIa	-	Cyclic peptide, large protein thermolabile	Enterocin As-48	[7,10]
IV	-	Consists of heat-labile peptides with a high molecular weight	Enterolysine A	[9]

## 3. Preservative Effects of Bacteriocin Produced by Lactic Acid Bacteria on Raw Meat Products

LAB is known for its capability to produce a variety of antimicrobial agents that can inhibit the growth of pathogenic bacteria. In 1988, the FDA approved the use of nisin and pediocin, a bacteriocin produced from *Lactococcus lactis* and *Pediococcus* sp. as preservatives for application in the food industry. Nisin and pediocin have been successfully commercialized widely [7]. In addition to nisin and pediocin, a bacteriocin from *Enterococcus* sp. namely enterocin has also gained significant academic interest following the research conducted on the effectiveness of antimicrobial agents produced by this species for use in food as a preservative. Ben Braïek et al. [17] stated that enterocin produced by *Enterococcus* sp. has high anti-listerial properties due to the bacteriocins produced by *Enterococcus* species being mostly classified as class III. It has a C-terminal disulfide bridge that stabilizes the posterior fold in the structure, which is crucial in enhancing the antimicrobial activity of the species [18]. In a study conducted by Fathizadeh et al. [19], recombinant bacteriocin, enterocin A and colicin E1 (ent A-col E1) exhibited antibacterial characteristics against both Gram positive and negative bacteria. Enterocin 12a produced by *E. faecium* was able to inhibit the growth of pathogens, such as *Salmonella enterica, Shigella flexneri, Vibrio cholerae, E. coli* and *L. monocytogenes* [20]. Several studies have reported the effectiveness of bacteriocin produced by LAB in inhibiting the growth of *L.monocytogenes* as shown in (Table 2). LAB are mainly from the genus of *Enterococcus* (*E. lactis* Q1, *E. lactis* 4CP3, *E. faecalis*), *Lactobacillus* (*L. paracasei*, *L. plantarum*, *L. sakei*, *L. reuteri*), and *Pediococcus*. Most of the bacteriocins produced by these LAB were able to inhibit the growth of *L. monocytogenes*. Based on Table 2, the treatment of *E. lactis* 4CP3 (enterocin A, B, P), and *E. faecalis* (enterocin AS-48) against *L. monocytogenes* resulted in growth inhibition activity as reported by Ben Braïek et al. [17,21] and Sparo et al. [22]. Meanwhile, enterocin P produced by *E. lactis* Q1 reportedly exhibited antimicrobial activity, as observed in the stunted growth of *L. monocytogenes* after 7 days of treatment as compared to the untreated sample [23]. Paracin C by *Lactobacillus paracasei*, Plantaricin (EF, W, JK, S) produced by *Lactobacillus plantarum* and bacteriocins produced by *Lactobacillus sakei*, *L. reuteri*, *L. plantarum*, *L. fermentum* inhibited the growth of *L. monocytogenes* while the treatment of Sakacin G produced by *Lactobacillus sakei* resulted in a decrease in the number of *L. monocytogenes* cells on roasted meat [24]. In addition, pediocin produced by *Pediococcus* sp. was found to exert broad spectrum antimicrobial activity against *L. monocytogenes* [25].

## 4. The Application of Enterocin on Raw Meat Products

Nowadays, the preservation methods in the food industry are evolving, with the use of bacteriocin aiding the process of preserving raw products. Bacteriocin is known for its capability to inhibit the growth of spoilage bacteria, such as *L. monocytogenes*, *Salmonella sp*., and *E. coli* in commercial food products so the quality can be maintained over a certain period. A newly reported byproduct rich in enterocin AS-48, and known to have a wide spectrum of antibacterial activity, might have good potential to be used as an additive since it achieved a good safety profile indicated by the negative result of the mutagenicity and genotoxicity assay test [29]. About 500 µL/animal/d of enterocin have been used as additives and were administrated in the drinking water of rabbits. As a result, the enterocin significantly affected the quality and mineral content of the rabbit meat, mainly iron and phosphorus [30]. There are several species of *Enterococcus* used as preservatives in raw products. For instance, the cell-free supernatant of *Enterococcus faecium* TJUQ1 combined with the bacterial cellulose of *Gluconabacter xylinus* forms a composite film, BC-E, which shows antibacterial activity against *L. monocytogenes* after being soaked and applied on ground meat [31]. Other examples were recorded as shown in (Table 3). There are several techniques for incorporating bacteriocin into food products: (1) inoculation of bacteria producing bacteriocin directly onto the meat or meat products as a starter or protective culture, (2) the use of purified or semi-purified cell-free supernatant directly as a food preservative, and (3) incorporation of purified and semi-purified bacteriocin and in packaging material [2,16].

Abts et al. [42] stated that enterocin is used as a food preservative through two methods: (1) direct inoculation of bacteria producing enterocin directly as a starter or protective culture, and (2) the use of purified or semi-purified cell-free supernatant. However, enterocin is often widely applied as a starter culture. For example, *E. faecium*, *E. mundtii*, and *E. classeliflavus* have been used as a starter culture in the production of fermented sausage [7,8,39]. As a result, *Enterococcus* sp. competes partially during the meat fermentation process, inhibiting the growth of *Listeria* sp. in the product [8].

Enterocin is also associated with several biochemical activities that stimulate aroma development through glycolysis, proteolysis, and lipolysis activities. In addition, it also plays a role in reducing the activity of metmyoglobin (MetMbO), which is an important mechanism for maintaining meat color [43]. Furthermore, enterocin also helps the degradation of stachyose and raffinose, the non-digestive oligosaccharides known as anti-nutrient factors [11]. The use of purified or semi-purified cell-free supernatant is also one of the methods often used for raw products, conferring the same benefits as that of the inoculation method in terms of inhibiting the growth of *L. monocytogenes*. This method is particularly useful in stimulating the formation of compounds that give aroma and taste to the product. However, this preservation method also has several disadvantages. While bacteriocin can inhibit oxidative rancidity due to damage that occurs in fats or oils, the production of unwanted flavors may also occur as a result of fat hydrolysis by lipase enzymes or from contaminating microorganisms [2].

Several researchers have suggested that the use of purified or semi-purified cell-free supernatants is suitable for application in food products, as it is more effective than the direct inoculation of the bacteriocin-producing bacteria. The latter may cause damage to the food in hostile environments [44]. During the purification process, all contaminants with low molecular weight are removed, leaving only the bacteriocin with a specific activity. The purification step allows for a more accurate determination of the biological activity of bacteriocin [42]. On the other hand, it has been reported in some cases that the use of cell-free supernatant on raw meat can potentially reduce the antimicrobial activity of bacteriocin due to the protein degradation that takes place when the supernatant is absorbed into the meat matrix [2]. Thus, Silva et al. [45] and, Borges and Teixeir [46] have suggested an alternative method by incorporating the purified or semi-purified bacteriocin in packaging material to increase the activity and stability of the bacteriocin in complex food systems. Referring to Table 3, enterocin A and B from E. faecium were incorporated into an alginate film, which is one of the packaging techniques used for fermented dried sausages, minced pork, and ham [36].

## 5. Effects of Enzyme, Temperature, and pH on the Activity of Enterocin

Characterization of bacteriocin is important to evaluate its effectiveness to be applied in the food industry. According to previous researchers, *E. faecalis* and *E. faecium* are the most commonly used bacteria from the genus *Enterococcus*, particularly in the food industry. The bacteria are used for the preservation of raw materials due to their high stability against extreme temperature and pH as compared to other species of *Enterococcus* as shown in (Table 4). The sensitivity of bacteriocin towards pH is diverse. The bacteriocin, known as enterocin As-48 produced by *E. faecalis* maintains its activity at pHs as high as 12 and temperatures of 121 °C for 15 min. Meanwhile, the activity of bacteriocin produced by *E. lactis* and *E. durans* was inhibited at 121 °C after 15 min. On the other hand, *E. mundtii*, which produces mundticin, can maintain its stability at 121 °C for 15 min; however, its activity is typically inhibited at pH 12. By referring to Table 4, *E. faecalis* and *E. faecium* are suitable for application on food products, such as raw meats and vegetables since they are stable at high temperatures and pH.

Bacteriocin produced by *Enterococcus* sp. as listed in Table 4 is typically sensitive to proteolytic enzymes, such as protease K and trypsin, which demonstrated the proteinaceous properties of the bacteria. Meanwhile, chymotrypsin, lipase, and catalase do not exert any effect on enterocin activity, indicating that the inhibition of bacterial growth is not due to the production of hydrogen peroxide [47]. Application of this proteolytic enzyme leads to protein degradation, and therefore, is safe for human consumption [11]. In the meantime, the loss of activity of bacteriocin depends on the formation of peptides and amino acid sequences.

According to Gao et al. [48] lowering the pH will gradually deactivate the growth of microorganisms. Most cationic bacteria will undergo cell lysis as a result of stimuli formed by negatively charged molecules found on the bacterial cell surface, such as lipopolysaccharide (LPS), lipoteichoic and teichoic acids. The findings demonstrate that the bacteriocin produced by *Enterococcus* sp. has a high resistance to extreme pH ranges and has the potential to be used in acidic and alkaline processed foods [49].

Temperature is crucial in ensuring the stability of bacteriocin activity. Based on Table 4, the activity of enterocin from *E. faecalis*, and *E. mundtii* is stable at a maximum temperature of 121 °C for 15 min while *E. faecium*, *E. durans*, and *E. lactis* could only withstand temperatures up to 100 °C for 30 min. Enterocin produced by *Enterococcus* is a heat-tolerant bacteriocin. The activity performed differs according to the species and molecular structure of the respective bacteriocin [32,49]. Some highly heat-sensitive bacteriocin lose their activity at 50 °C due to the loss of their original secondary and tertiary structure as a result of denaturation [50]. The resistance of *Enterococcus* at pasteurization temperature and its adaptability to substrate and growth conditions demonstrates its potential application in food products.

**Table 4:** Activity of bacteriocin produced by *Enterococcus* sp. against enzymes, temperature, and pH.

Strain	Bacteriocin	Stability															References
		Enzyme					Temperature (°C/min)				pH						
		*Proteases K*	Trypsin	Chymotrypsin	Lipase	Catalase	65 °C/30 min	80 °C/30 min	100 °C/30 min	121 °C/15 min	2	4	6	8	10	12	
*E. faecalis*	Enterocin As-48	−	−	+	+	+	+	+	+	+	+	+	+	+	+	+	[32,51,]
*E. faecium*	Enterocin A and B	−	−	+	+	+	+	+	+	−	+	+	+	+	+	+	[38]
*E. durans*	Enterocin L50A- like bacteriocin and L50B, Durancin GL	−	−	+	+	+	+	+	+	−	+	+	+	+	−	−	[32,34,35]
*E. mundtii*	Mudticin	−	−	+	+	+	+	+	+	+	+	+	+	+	+	−	[41]
*E. lactis*	Enterocin A, B, and P	−	−	+	+	+	+	+	+	−	+	+	+	+	+	−	[17,21]

## 6. Various Methods Used in Raw Meat Preservation

Preservation of raw meat is crucial to ensure that the quality of the product is maintained throughout the long period of transportation and commercialization without damaging the texture, color, and nutritional value of the food. Currently, there is high demand for food that is free from synthetic ingredients. Consequently, biopreservation techniques have started to gain significant interest for application in the food industry. One of the challenges faced by the butchers and sellers is to maintain the quality of raw products while ensuring that the products are free from unwanted microbial growth. This is particularly critical for global suppliers who import raw materials to be commercialized around the globe [52]. Therefore, various methods have been invented to solve the problem. These methods are categorized into four categories based on the use of (1) natural biopreservatives and chemicals, (2) refrigeration technique, (3) innovative packaging, and (4) non-thermal processing (Table 5).

### 6.1. Use of Natural Bio Preservatives and Chemical Preservatives

#### 6.1.1. Biopreservation

Biopreservation is a method of preserving food by using microorganisms as a protective culture. This method is performed by inoculating the food with selected lactic acid bacteria to inhibit the growth of spoilage bacteria [54]. The demand for high-quality processed foods that are safe to eat has encouraged the application of this method in the food industry. For example, bacteriocin known as nisin is one of the food preservatives produced from lactic acid bacteria, which have been commercialized and approved by the FDA [66]. According to Singh [1] bacteriocin is considered a good preservative substance as it does not stimulate the immune response (non-immunogenic) and has high thermal resistance, as well as extensive antimicrobial activity.

In the meat-based industry, bacteriocins, such as nisin, enterocin As-48, enterocin A and B, sakacin, leucocin, and pediocin are highly effective at inhibiting the growth of *L. monocytogenes* and other pathogenic bacteria [67]. However, the antimicrobial activity of nisin is very limited when applied to meat products because of its low solubility rate, besides the potential destruction of the enzyme activity related to inhibition of pathogenic bacteria [2]. Thus, enterocin As-48, enterocin A and B, sakacin, leucocin, and pediocin are more effective for meat preservation as the majority of these bacteriocins belong to class II, known to inhibit *L. monocytogenes* [68,69]. The ability of bacteriocin to inhibit the growth of pathogenic bacteria in the food is attributable to its ability to form electrostatic interaction with the negatively charged phosphate groups in the cellular membrane of the targeted bacteria. This interaction occurs with initial bonds formed on the cell membrane, followed by the formation of pores that induces cell lysis [1].

#### 6.1.2. Chemical Preservation

The chemicals that are commonly added to the food to maintain the nutritional value and quality of the food include chlorides, nitrites, sulfides, and organic acids. The combination of this method with freezing techniques often provides the best protection to the meat product. The storage methods alone are not effective in preventing oxidative disorders and inhibiting microbial or enzyme activity. On the other hand, the combined methods can increase the stability, maintain the freshness and nutritional value as well as the quality of the product [57].

Nitrites are often used in the process of meat and cheese production as an additive to increase the stability of the product. European Union under Commission Regulation (EU) No 1129/2011 has allowed the use of nitrites, such as sodium nitrite, potassium nitrite, and sodium nitrate in food, However, the use of nitrate is limited to 150 mg Kg^−1^ for meat processing and 100 mg Kg^−1^ for meat sterilization processes [70]. Nitrate can maintain the quality of raw meat through the formation of bonds between the nitrate oxide (NO) in the nitrate and the ferum ion (Fe^2+^) at the center of the myoglobin porphyrin ring system, resulting in the formation of unstable nitrosomyoglobin. The nitrosomyoglobin may be converted into a characteristic red pigment in meat products called nitroso-myochromogen through a heating process in an acidic state (fermented meat) [71]. However, this method has become a concern among buyers in terms of the potential negative impact on human health and worse, it can be fatal. For example, nitrite is known to potentially inhibit the growth of *Clostridia* sp. in meat products but may also react with the secondary amines to form carcinogenic nitrosamine, which is harmful to the fetus [46,72]. As for organic acids, they act as antioxidants and prevent damage to meat products. This acid is easy to find and typically non-toxic, making it suitable for application in raw meat processing [73]. Thus, FDA has classified organic acids as a Generally Recognized as Safe (GRAS) for application in meat products, with no known side effects reported [57].

### 6.2. Refrigerator

#### 6.2.1. Chilling

A traditional cooling temperature is usually between 0 °C and 7 °C. This method is used to reduce microbial growth in food. Chilling is very important in maintaining meat hygiene, safety, quality, and appearance. Chilling by air can lower the temperature on the surface of the carcass and increase its drying rate. As a result, the microbial growth in meat products can be reduced [40].

#### 6.2.2. Super Chilling

Super chilling is a process that involves a change in temperature between conventional cooling and freezing. This method occurs through two stages namely (1) initial freezing, and (2) heat absorption. At the initial freezing, ice of approximately 1–33 mm in size is formed when the temperature of the food is within 1–2 °C. Thereafter, the heat on the outer layer of the food will be absorbed, promoting equilibration in the temperature between its inner and outer layers during the storage and distribution process [74,75]. There are several benefits in applying this method that include the inhibition of unwanted microbial growth and extension in the shelf life of the product by at least 1.4 to 4 times as compared to that of traditional methods, However, this method was found effective only on seafood and non-meat products as it often results in the loss of water content in meat [74].

#### 6.2.3. Freezing

Freezing is one of the methods commonly used in ensuring that perishable food, such as raw food, lasts longer. The ideal storage temperature for frozen meat is −55 °C, which maintains the quality of the food. This freezing process allows the temperature of the meat to drop to the desired temperature causing the formation of ice crystals in the meat. The formation of these ice crystals prevents the changes in the texture and taste of the meat [58] since the low temperature can minimize enzyme reaction processes, reduce oxidative rigidity, ice recrystallization, and reduce the damage rate. Note that some products may still be damaged during storage [40,76].

### 6.3. Packaging

#### 6.3.1. Vacuum Packaging

Vacuum packaging is a method of modified atmosphere packaging (MAP), which involves the removal of air from gas-tight packaging before an immediate sealing. Air is an important factor in the regulation of microbial growth in food products. Therefore, air should be removed from the packaging as it supports the growth of lactic acid-producing bacteria, which are capable of producing carbon dioxide which is important in preventing the growth of food spoilage bacteria. As a result, the shelf life of the product can be increased [77]. The carbon dioxide emission will increase rapidly by 10 to 20% within the first 4 h of the process, and subsequently, reach a maximum level of up to 30%. In the meantime, the oxygen level will be reduced to 1 to 3% as a result of the enzyme activity that occurs in the meat [60]. According to Stasiewicz et al. [78], sausages that were subjected to this process have a more palatable taste compared to that of the MAP.

However, there are some negative opinions from researchers regarding the use of this packaging method. The use of vacuum packaging for food products is constrained by the fact that the red pigment of the fresh meat will change into dark purple after the packaging process (Deoxymyoglobin), which influences the buyer’s decision in buying the product [79]. Furthermore, this method also affects the water content in meat as compared to the MAP method due to the high pressure and long storage period.

#### 6.3.2. Modified Atmosphere Packaging (MAP)

This process involves the removal of gas in the environment and replacing it with other gases, such as oxygen, carbon dioxide, and nitrogen. Oxygen acts as an inhibitor for the growth of anaerobic bacteria and can maintain the myoglobin form. Meanwhile, carbon dioxide can inhibit the growth of aerobic spoilage bacteria and nitrogen can maintain the shape of the package [52]. These gases can be used separately or in combination to achieve optimal effect. In addition, several parameters influencing the effectiveness of MAP have been identified, such as the film permeability to oxygen, carbon dioxide, water vapor, film thickness, the surface area of the packaging, and free volume in the package [80].

#### 6.3.3. Active Packaging

Active packaging is a type of food packaging method that has an additional function other than inhibiting microbial growth. This method involves absorbing the chemicals derived from food or the environment surrounding the package and secreting substances that include the preservative, antioxidants, and flavoring compounds into the food or its surroundings [81]. There are two types of active packaging, namely the active absorbent system (absorber) and active release system (release). Examples include oxygen absorption and the use of carbon dioxide emitters and absorbers, moisture absorbers, and antimicrobials. Compounds, such as carbon dioxide, oxygen, ethylene, moisture, or odors present in the product and its surrounding will be released. Then, other compounds will be mixed into the packaging of the product.

Antimicrobial packaging is a system in which the growth of microorganisms is inhibited so the shelf life of the food can be extended by using natural antimicrobials. Most antimicrobial packaging is invented in the form of edible films or coatings [82]. Various antimicrobial compounds are incorporated in this system, such as organic acids, chitosan, bacteriocin, EDTA, lysozyme, essential oil, cinnamon, and many others as they are volatile and have great antimicrobial activity [82,83]. These antimicrobial compounds are often used by associating them with the film coating and various antimicrobial agents to further enhance the effectiveness of the antimicrobial activity of the agents that are already present in the matrix. The compounds will be released to the food surface either through migration or evaporation headspace [83]. Through this combination, the antimicrobial agents can be released gradually to control the level of antimicrobial activity and sensory changes in food products [84].

### 6.4. Non-Thermal Processing

#### 6.4.1. Ionizing Radiation

Radiation is a preservation process using radiation, such as gamma, infrared, and UV on the product. It is also known as cold sterilization. As a result of this radiation energy, the chemical bonds in the microbial DNA molecules are broken, stunting the growth of the microorganism [85]. The food processed using ionizing rays have distinct qualities compared to those of traditional methods. This process is carried out by subjecting the product to ionizing radiation. The chemical bonds present in the product will absorb the energy generated from the radiation, prompting some of the bonds to break down, which leads to the production of reactive and unstable free radicals. Subsequently, the free radicals will form a new bond from the adjacent compound, leading to the formation of radiolysis compounds [63].

#### 6.4.2. High Hydrostatic Pressure

High hydrostatic pressure is another example of the non-thermal process, which is used to deactivate microbes and reduce the chemical reaction in food at a pressure of 100 MPa (986.9 atm/1019.7 kgf/cm^2^) [65]. This process is called batch processing in which the packaged food is treated in a chamber surrounded by a pressure transmitter fluid. A semi-continuous system has also been invented for pumpable food, whereby the food is compressed without the use of a container and packaged aseptically [86].

This method allows for microbial inactivation in a food product through disruption of the cell morphology and some susceptible cellular components, such as plasma membranes, ribosomes, and enzymes, including those that are essential for DNA replication and transcription [57]. The study conducted by Bover-Cid et al. [87] showed that at a high hydrostatic pressure of 600 MPa, the growth of *L. monocytogenes* was reduced by 5 logs in dry-cured ham after a 10-min treatment. Through this study, they confirmed that pressure, time, and temperature are interrelated with each other in inhibiting the growth of *L. monocytogenes*. Apart from inhibiting microbial growth, high hydrostatic pressure also increases the tenderness of the meat and the thermal gel capacity or actomyosin in the meat. Such effects are achieved due to the changes in the tertiary structure of myosin, causing hydrophobic and sulfhydryl bonds to be exposed, which strengthen the gel, especially at low salt concentrations [88].

## 7. Comparison of Preservation Method by Biopreservation and Commercial Techniques

Generally, each technique used for food preservation has its advantages in terms of inhibiting microbial growth and maintaining the quality of the food product. Similarly, each method also has its drawbacks as shown in Table 5. Table 6 shows a comparison between biopreservation and commercial techniques used in the preservation of raw meat in terms of cost, effectiveness, control range, type of food products, and methods and materials used.

Overall, biopreservation techniques are known for their low cost and effectiveness in maintaining the intrinsic characteristics of the product, although the application is quite limited. Meanwhile, commercial preservation has been widely applied despite the high operating cost and the potential changes in the intrinsic characteristics of the food product. Biopreservation is known as a preservation technique applied to help extend the shelf life of food products by using beneficial bacteria namely LAB, which produces a broad-spectrum antimicrobial agent called bacteriocin [1]. Meanwhile, commercial techniques often involve the use of advanced technologies, such as high hydrostatic pressure, ionizing rays, vacuum and active packaging, modified atmosphere packaging (MAP), and freezing technology. Therefore, the cost of commercial techniques is generally higher than that of the biopreservation technique and typically requires skilled manpower to operate. For example, Yordanov and Angelova [89] stated that high-pressure technology is currently more expensive than other processing technology because it involves the use of high-pressure vessel components and covers, temperature control devices, and material handling systems. On the other hand, the application of biopreservatives in food products does not affect their intrinsic properties [4,90]. Intrinsic properties are divided into several categories namely water content, nutrients, pH, redox potential, and biological structure [91]. The use of commercial techniques in preserving food is less effective because of the potential effect on the intrinsic properties of food products. This is supported by Lilian et al. [75] who stated that supercooling from freezing technology led to water loss from the food product, causing changes in texture on the products. Additionally, Scetar et al. [61], and Mathew and Jaganathan [60] have asserted that the lipid and protein oxidation that takes place in a vacuum and active packaging techniques often result in a significant change in the color of the food, particularly that of raw meat.

Bacteriocin is known to exhibit extensive antimicrobial activity to inhibit the growth of pathogens. Nowadays, the commercial use of bacteriocin produced by Gram-positive bacteria, such as LAB is gaining popularity because of its inhibitiory activity against the growth of foodborne pathogens, especially Gram-positive pathogens [1,11]. However, Gram-negative pathogens are less sensitive to bacteriocin produced by Gram-positive bacteria due to the presence of an outer layer membrane that effectively protects the pathogens [92]. Commercial techniques, such as high hydrostatic pressure and ionizing rays can disrupt the stability of the outer membrane layer of Gram-negative bacteria and inhibit their growth. However, this method is not applicable for Gram-positive bacteria as it requires higher pressure to be inactivated [93]. Therefore, the combination of biopreservative application with other commercial techniques can enhance inhibitory activities against foodborne pathogens. For instance, the ability of biopreservatives to inhibit the pathogen of Gram-positive bacteria can be exploited via the use of high hydrostatic pressure [92]. Moreover, based on the study of Babich et al. [94] fruit samples that had been treated with biopreservatives and packaged using modified atmosphere packaging (MAP) produced relatively good microbiological results after storage for 25 days compared to not combining the techniques. Biopreservatives are commonly applied in food, such as dairy products, meat, and vegetables. For example, *Lactococcus lactis*, which produces bacteriocin known as nisin, is used as a starter culture in cheese making to inhibit the growth of *L. monocytogenes* [45]. Meanwhile, *E. faecium* is often used in meat-based products, such as fermented sausages, minced pork, and ham [36]. The products that are often subjected to preservation using commercial techniques include dairy products, meat, vegetables and fruits, fresh juices, vegetable purees, and jams [93]. The high hydrostatic pressure technique is typically used to improve the coagulation properties of milk and retain the moisture in fresh cheese. It is also used to preserve and retain the color and stability of fruit juices during the storage process [95]. On the other hand, freezing technology allows for perishable foods, such as meat to last longer [58].

## 8. Conclusions

The preservation effects of bacteriocin produced by lactic acid bacteria of the genus *Enterococcus* are discussed in this review. The use of natural food preservation aids in the reduction of the use of chemical ingredients that may be damaging to consumer health. This review compiles various methods of food preservation techniques that can be applied at multiple scales. The application of biopreservatives combined with other techniques should be investigated further in future research to determine the most effective way in preserving food using via biopreservation techniques. The findings will benefit the food industry, in addition to enhancing the safety and quality of food products that are delivered to consumers.

## Figures and Tables

**Table 2:** Bacteriocin produced by lactic acid bacteria tested on raw meat.

Lactic Acid Bacteria	Bacteriocin	Inhibitory Effect	References
*Enterococcus lactis* Q1	Enterocin P	*L. monocytogenes* cell decreased to 6.47 ± 0.30 log unit after 7 days as compared to control that was not treated with *E. lactis* (7.25 ± 0.35 log unit after 14 days) and maintained until 28 days in the fridge.	[23]
*Enterococcus lactis* 4CP3	Enterocin A, B, and P	The growth of listerial was completely inhibited from day 14 until 28.	[17,21]
		The inhibition of *L. monocytogenes* growth on the rabbit meat during cold storage was detected on day 28.	
*Enterococcus faecalis*	Enterocin AS-48	There was no detection of *L. monocytogenes* growth on the beef after 24 h treated with *E. faecalis.*	[22]
*Lactobacillus paracasei*	Paracin C	The growth of pathogenic bacteria was inhibited, and the color of the meat was retained until day 15.	[26]
*Lactobacillus plantarum*	Plantaricin EF, W, JK and S	The growth of both spoilage bacteria was inhibited by *L. plantarum* until day 15 at 22 °C.	[27]
*Lactobacillus sakei*	Sakacin G	The application of *L.sakei takes* on roasted meat resulted in a decrease in the number of *L. monocytogenes* cells. Meanwhile, for chicken breast, the inhibition effect depleted.	[24]
*Lactobacillus sakei, L. reuteri, L. plantarum, L. fermentum*	Bacteriocins	The formation of the inhibition zone after the treatment of bacteriocin demonstrated the growth inhibition of *L. monocytogenes*.	[28]
*Pediococcus* sp.	Pediocin	Pediocin and pediocin-like bacteriocins exerted a broad spectrum of activity against *L. monocytogenes* through the formation of pores in the cytoplasmic membrane and cell membrane dysfunction.	[25]

**Table 3:** The types of enterocin produced by *Enterococcus* sp. used in raw meat products.

Producer Strain	Types of Enterocin	Product	Additional Technique Used	Targeted Pathogenic Bacteria	References
*E. faecalis*	Enterocin As-48	Fermented sausage	Mixed with bacteriocin/chemical preservatives	*L. monocytogenes*	[16,32]
*E. durans*	Enterocin L50A-like bacteriocin & L50B (Dur 152A)	Ham	Semi-purified bacteriocin/anti-listerial protection	*L. monocytogenes*	[33,34,35]
*E. faecium*	Enterocin A and B	Fermented dried sausage, minced pork, and ham	Applied on the surface of meat/alginate film/high hydrostatic pressure	*Listeria* spp, *L. sakei*	[16,36,37,38]
*E. classeliflavus*	Enterocin 416kk1	Cacciatore (Italian sausage)	Starter culture/low-density polyethylene film	*L. monocytogenes*	[39,40]
*E. mundtii*	Mundticin	Fermented fish and seafood, sausage	Starter culture/chitosan	*L. monocytogenes*	[7,41]

**Table 5:** Various methods used in preserving meat.

Category	Method	Description	Advantages	Disadvantages	References
**Application of chemical and organic**	Biopreservation	The application of bacteriocin produced by lactic acid bacteria with a specific antimicrobial activity	Helps in extending the shelf life of the product and subsequently, improves the unique taste and texture of the product	Sensitive to food enzyme, low level of solubility, and easy to be absorbed	[1,53,54]
	Chemical preservation	The process of controlling spoilage microorganisms using antimicrobial compounds, such as chlorides, nitrites, sulfides, and organic acid	Adds flavor and extends the shelf life of the product	Causes change in color, bad odor, and long-term harmful effects on human health	[55,56,57]
**Refrigeration**	Chilling	Reducing the microbial reproduction at temperatures below its optimal temperature for growth by 2–5 °C.	Ensures the cleanliness and safety of the meat, prolongs the shelf life, and retains the nutritional quality	An increase in air velocity or a decrease in temperature affects the cooling time, as well as the slow heat-release throughout the meat tissues	[40]
	Super Chilling	The process of freezing the water content in a particular product at 1–2 °C.	Prevents the growth of microbes, reduces labor cost, and maintains the product weight	Complex calculations are required to determine effective heat transfer as well as temperature control	[40,58]
	Freezing	The temperature of −55 °C is the ideal storage condition to freeze the product to ensure that the product quality is maintained	Helps in inhibiting microbial growth and stops the enzyme activity	Deformation of product occurs due to the cryogenic process, affecting the commercialization process	[40,57,59]
**Packaging**	Vacuum packaging	The use of packaging for raw meat includes the use of air-permeable packaging, low oxygen vacuum, low MAP oxygen with anoxic gas, and high MAP oxygen	This process can prevent the product from being contaminated and reduced in weight, increase the tenderness of the meat, and maintain the color of oxymyoglobin in meat	The active compound is unstable during the process of spraying the packaging material and the mass is too small to be transferred to the product	[52,60,61]
	Modified atmosphere packaging (MAP)				
	Active packaging				
**Non-thermal processing**	Ionizing radiation	Radiation (gamma, infrared, UV) is applied to the product, causing the fragmentation of DNA molecules in the microbes	Effective in inactivating the growth of bacteria on the product and does not change chemically	Discoloration of meat may occur	[40,62,63]
	High hydrostatic pressure	This process is non-thermal, used to deactivate microbes and reduce the chemical reaction in food using high-pressure technology at 100 MPa (986.9 atm/1019.7 kgf/cm^2^).	Eliminates spoilage bacteria without changing the color, taste, texture, and moisture of the product and increase the shelf life as well as the tenderness of the meat	Intrinsic and extrinsic factors affect the pressure resistance	[64,65]

**Table 6:** Comparison of preservation method by biopreservation and commercial techniques.

Preservation Technique	Biopreservation	Commercial Techniques
Cost	Low in cost as there is no need for advanced equipment	High in cost due to the use of advanced technology and skilled manpower
Effectiveness	More effective as it does not affect the intrinsic properties of the product	Less effective as it affects the intrinsic properties of the product
Range of control of food pathogens	More efficient against food pathogen of Gram-positive than Gram-negative	Less efficient against food pathogen of Gram-positive than Gram-negative
Type of product/foods	Limited application to dairy products, meat, and vegetables	Wide application for dairy products, meat, vegetables and fruits, fresh fruit juices, vegetable purees, and jams
Method/materials used	Bacteriocin from lactic acid bacteria (LAB)	High hydrostatic pressure, ionizing radiation, vacuum and active packaging, modified atmosphere packaging (MAP), and refrigeration.

## Data Availability

Not applicable.
